# The Spatio-Temporal Dynamics of Phoneme Encoding in Aging and Aphasia

**DOI:** 10.1523/JNEUROSCI.1001-25.2025

**Published:** 2025-12-29

**Authors:** Jill Kries, Maaike Vandermosten, Laura Gwilliams

**Affiliations:** ^1^Department of Psychology, Stanford University, Stanford, California 94305; ^2^Wu Tsai Neurosciences Institute, Stanford University, Stanford, California 94305; ^3^Department of Neurosciences, Experimental Oto-Rhino-Laryngology, KU Leuven, Leuven 3000, Belgium; ^4^Leuven Brain Institute, KU Leuven, Leuven 3000, Belgium; ^5^Leuven Interdisciplinary Language Institute, KU Leuven, Leuven 3000, Belgium; ^6^Stanford Data Science, Stanford University, Stanford, California 94305

**Keywords:** aphasia, EEG, language processing, phonetic features, speech encoding

## Abstract

During successful language comprehension, speech sounds (phonemes) are encoded within a series of neural patterns that evolve over time. Here we tested whether these neural dynamics of speech encoding are altered for individuals with a language disorder. We recorded EEG responses from the human brains of 39 individuals with post-stroke aphasia (13♀/26♂) and 24 healthy age-matched controls (i.e., older adults; 8♀/16♂) during 25 min of natural story listening. We estimated the duration of phonetic feature encoding, speed of evolution across neural populations, and the spatial location of encoding over EEG sensors. First, we establish that phonetic features are robustly encoded in EEG responses of healthy older adults. Second, when comparing individuals with aphasia to healthy controls, we find significantly decreased phonetic encoding in the aphasic group after a shared initial processing pattern (0.08–0.25 s after phoneme onset). Phonetic features were less strongly encoded over left-lateralized electrodes in the aphasia group compared to controls, with no difference in speed of neural pattern evolution. Finally, we observed that healthy controls, but not individuals with aphasia, encode phonetic features longer when uncertainty about word identity is high, indicating that this mechanism—encoding phonetic information until word identity is resolved—is crucial for successful comprehension. Together, our results suggest that aphasia may entail failure to maintain lower-order information long enough to recognize lexical items.

## Significance Statement

This study reveals robust decoding of speech sound properties, so-called phonetic features, from EEG recordings in older adults, as well as decreased phonetic processing in individuals with a language disorder (aphasia) compared to healthy controls. This was most prominent over left-hemispheric electrodes. Additionally, we observed that healthy controls, but not individuals with aphasia, encode phonetic features longer when uncertainty about word identity is high, indicating that this mechanism—encoding phonetic information until word identity is resolved—is crucial for successful language processing. These insights deepen our understanding of disrupted mechanisms in a language disorder and show how the integration between language processing levels works in the healthy aging and neurotypical brain.

## Introduction

Comprehending natural speech entails rapidly converting continuous acoustic input into discrete units, such as phonemes and words (Hickok and Poeppel, 2007). Gwilliams et al. (2022) demonstrated that the spectrotemporal properties of phonemes, known as phonetic features, are dynamically encoded in the brain. This coding scheme facilitates integration with higher-level language representations, such as syntactic or semantic features. Individuals with language disorders such as aphasia can have deficits in phoneme identification (Kries et al., 2023) or phonological processing (Blumstein, 1998), among other difficulties. With the hierarchical dynamic coding (HDC) framework (Gwilliams et al., 2025), we have a unique opportunity to investigate the neural dynamics of speech representations in individuals with aphasia (IWA). Here, we investigate the effects of aphasia on phonetic feature encoding.

When healthy young adults listen to speech, phonetic features are encoded in auditory cortex, including the transverse and superior temporal gyrus and sulcus (Mesgarani et al., 2007; Wilson et al., 2018; Levy and Wilson, 2019; Yi et al., 2019). Based on magnetoencephalography (MEG) data during story listening, Gwilliams et al. (2022) showed that the spectrotemporal properties of phonemes are encoded for around 0.3 s, outlasting the duration of the phoneme input, and that the encoded information is passed to a new neural ensemble every 0.08 s, which is proportional to the average phoneme duration in the stimulus. Phonetic features are thus encoded in a dynamic neural pattern, whereby different neural ensembles are recruited across time, i.e., HDC (Gwilliams et al., 2025). This means that rather than a one-to-one mapping between neural ensemble and feature encoding, different ensembles are activated in sequence. This allows a running history of three phonemes being encoded in parallel, while also tracking the relative order that they were heard.

Aphasia is often considered a phonological, semantic or syntactic disorder due to common symptoms like word-finding difficulties or incomplete sentences (Harnish, 2018; Zhang and Hinzen, 2022). However, recent work suggests that processing issues in aphasia can also be related to more low-level speech processing, i.e., encoding of speech amplitude fluctuations and phoneme identification (Kries et al., 2023, 2024). Some language difficulties may thus arise from an impoverished representation of speech sounds. In fact, Kries et al. (2023) found that IWA’s performance at discriminating different slopes in amplitude rise predicted phonological performance, suggesting that low-level impairments may propagate to higher-level processes. Building upon this prior work, we aim to assess whether difficulties in phoneme processing (and more generally lower-level processing) in aphasia may stem from challenges in dynamic coding of phonetic features.

**Figure 1.**
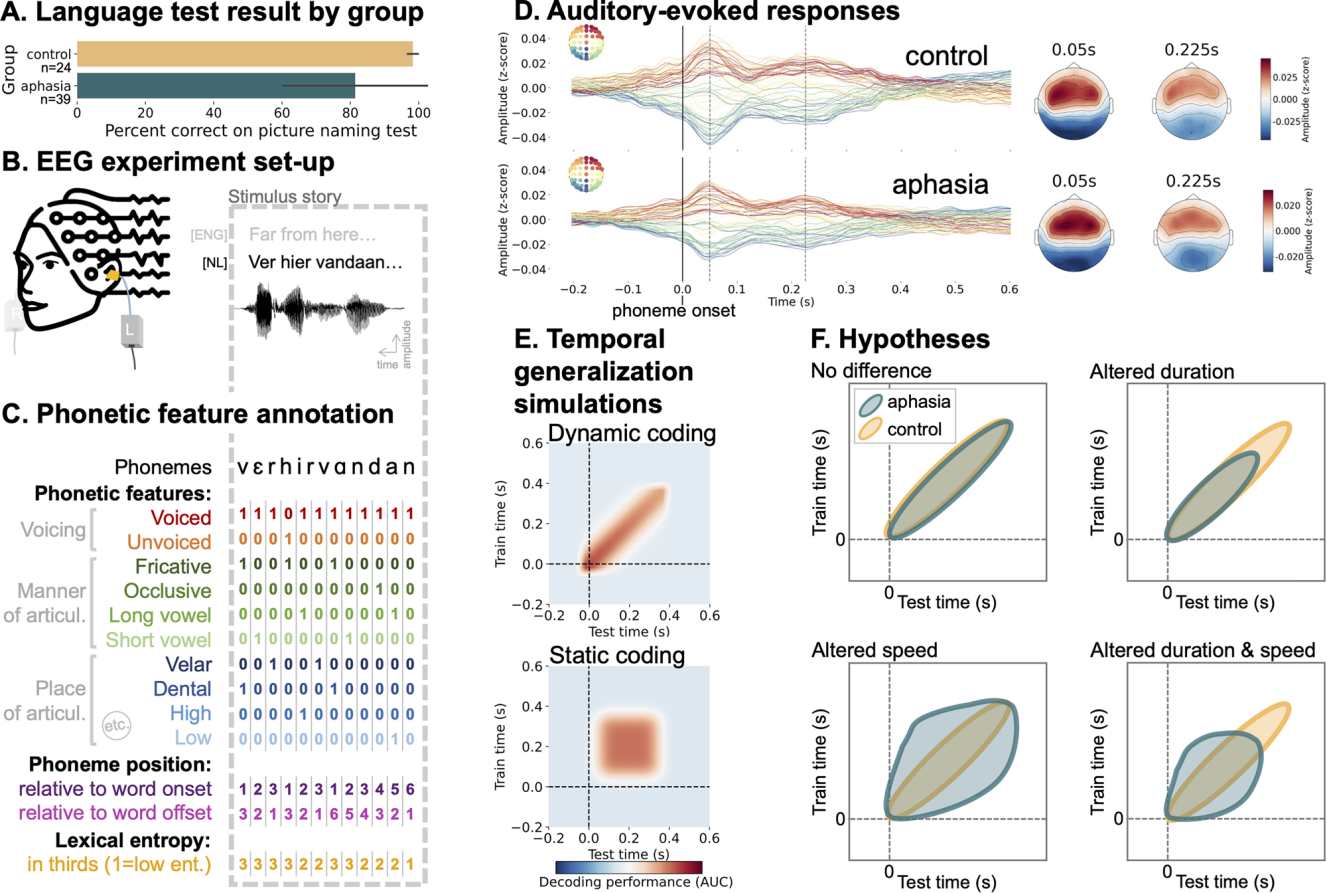
Experimental set-up, schematic of analytic approach, auditory-evoked responses by group and hypothesis illustration. ***A***, Mean and standard deviation for the picture naming test scores (in percentage) by group. ***B***, The experimental protocol consisted of participants listening to a 25 min-long narrative in Flemish while 64-sensor-EEG data was being recorded. The audio was presented bilaterally. The first 3 words and their acoustic waveform are depicted, with an English translation. ***C***, The narrative was annotated for 18 phonetic features (not all illustrated) with across-feature time alignment. Moreover, the phonemes were subset by position within word and by lexical entropy (entropy values were categorized into thirds). ***D***, The EEG data was epoched around the phoneme onsets. This plot displays the timecourses of the auditory evoked response for all 64 EEG sensors on the left and the topographies at 0.05 and 0.225 s after phoneme onset on the right, for the control group and aphasia group separately. ***E***, Temporal generalization (TG): Training a decoder at each time slice and testing each of these decoders across all possible time slices allows to investigate the encoding duration (matrix diagonal) and generalization (width of significant cluster). The top plot shows a dynamic encoding pattern wherein the encoded information evolves across temporal decoders, i.e., the way that information is encoded changes over time. The bottom plot shows a static encoding pattern wherein the encoded information is maintained. ***F***, We hypothesized that the aphasia group would show a different encoding pattern than the healthy control group. The top right graph illustrates what the TG pattern would look like if individuals with aphasia had a shorter encoding duration than controls. The bottom left graph shows what we would expect to see if individuals with aphasia had a slower speed of evolution of encoding than controls, i.e., longer generalization. The bottom right graph displays what we would expect to observe if both duration and generalization of encoding would be altered in aphasia.

Gwilliams et al. (2022) used MEG recordings to investigate phonetic encoding during a 2-h story listening task in younger adults. By contrast, we are using an available electroencephalography (EEG) dataset of IWA and healthy age-matched controls listening to natural speech for 25 min (Fig. 1*B*; Kries et al., 2024). EEG is more feasible than MEG for post-stroke or older adults because of its accessibility in clinical settings. Moreover, shorter neural recordings are desirable to maintain optimal attention levels. Therefore, our first research question examines whether the robust phonetic decoding observed by Gwilliams et al. (2022) in younger adults can be replicated with (1) EEG data, (2) shorter recording duration, and (3) in older adults.

The second research question examines the dynamics of phonetic encoding in IWA compared to healthy controls. Specifically, we wanted to know whether IWA would show changes in phonetic encoding duration, changes in speed of neural pattern evolution, or a mix of both (Fig. 1*F*). We hypothesized a slower speed of neural pattern evolution in aphasia, because that would imply representational overlap between neighboring phonemes and might explain symptoms observed in aphasia, such as phonemic paraphasias. Given the structural and functional neural changes following stroke-induced aphasia (Wilson et al., 2023), we also test for differences in topography of neural activity during phonetic encoding between IWA and healthy controls.

## Materials and Methods

### Participants

Thirty-nine IWA in the chronic phase after stroke [≥6 months after onset; 13♀/26♂; age in years: mean (standard deviation) = 69.5(12.4)] and 24 demographically-matched healthy control participants [8♀/16♂; age in years: mean (standard deviation) = 71.5(7)] listened to a narrative for 25 min (in their native language, Dutch) while EEG data was recorded (Fig. 1*B*). IWA were recruited at the university hospital in Leuven and via advertisement, see Text S11 for more information. All aphasia participants in the used dataset had clinically diagnosed post-stroke aphasia in the acute phase after stroke (0–14 days post-stroke). We only included IWA that had no formal diagnosis of a psychiatric or neurodegenerative disorder and that had a left-hemispheric or bilateral lesion. All aphasia participants were tested in the chronic phase after stroke [time since stroke onset in months (median (range)): 16.1 (6–126.1)], after spontaneous recovery and therapy. About 37/39 participants (95 %) still had residual language impairments at the moment of data collection, as determined by clinical evaluation and evidenced by continued attendance at speech therapy sessions.

The aphasia sample was checked for language impairments at the moment of data collection using two standardized diagnostic aphasia tests, i.e., the diagnostic test ScreeLing (Visch-Brink et al., 2010) and the Dutch picture naming test (NBT; Van Ewijk et al., 2020), using the same procedure as reported in Kries et al. (2024; Fig. 1*A*; Table S2). These tests reflect aphasia severity (also see Text S4). On both tests, the aphasia group had significantly lower results than the control group [ScreeLing cut-off threshold: 68/72 points; NBT cut-off threshold: 255/276 points; ScreeLing (mean (standard deviation)): aphasia = 61.5(9.4), control = 69.9(2.5), *p* < 0.001; NBT (mean (standard deviation)): aphasia = 224.7(58.1), control = 271.3(4.1), *p* < 0.001; Table S1 and Text S2]. Moreover, all participants completed further behavioral tasks assessing: hearing (pure tone audiogram), rise time discrimination, phoneme identification, phonological and semantic word fluency, cognition (total score of attention, executive function, and memory tasks; Huygelier et al., 2019), self-reported alertness and fatigue, story comprehension question accuracy (Kries et al., 2024). More details on these tasks and the group comparisons are available in the supplementary materialof this paper (Text S4). Eighty-two percent of the aphasia group showed deficits at either one of the two phonological tasks that we administered (i.e., phoneme identification task and phonology subtest of the ScreeLing; Text S4).

Throughout the paper, we are referring to healthy control participants and older adults interchangeably given that these participants are on average 71 years old, with a range of 58 to 87 years of age. All participants were Dutch native speakers from Flanders, Belgium. Age, sex, education, handedness, and multilinguality did not differ between groups (Table S1; Kries et al., 2024). Demographic information was acquired via a self-reported questionnaire. Handedness was assessed via the Edinburgh Handedness Inventory (Oldfield, 1971). Details about the stroke in IWA, i.e., time since stroke onset, stroke type, occluded blood vessel, lesion location, and speech-language therapy, can be found in Table S2 and Text S3. To visualize the damaged brain tissue of IWA, a lesion overlap image was created (Fig. S1 and Text S1). Informed consent was obtained from all participants for the recruitment via screening and for the data collection in the chronic phase. The study received ethical approval by the medical ethical committee of KU Leuven and UZ Leuven (S60007) and is in accordance with the declaration of Helsinki.

### Experimental design

The EEG measurements were conducted in a soundproof room with a Faraday cage using a 64-channel EEG system (ActiveTwo, BioSemi) at 8,192 Hz sampling frequency. Participants listened to a 25-min story, The Wild Swans written by Hans Christian Andersen, narrated by a female Flemish-native speaker. The stimulus story was specifically tailored to the aphasia population by choosing a fairytale as stimulus that consists of simple language. The narrative consisted of 13,560 phonemes, which had a median length of 0.070 s (min = 0.029 s; max = 0.359 s), and 3,002 words. Stimuli were presented binaurally via shielded insert earphones at 60 dB SPL (a weighted). Participants were asked five yes/no and five multiple-choice questions about the story content throughout to ensure attentiveness (see Text S4 for more information).

### EEG signal processing

The EEG signal processing was conducted using MATLAB [version 9.1.0.441655 (R2016b)]. Eye movement artifact removal was employed using the multichannel Wiener filter (Somers et al., 2018). Next, the EEG data was referenced to the common average and then downsampled to 512 Hz. Then, the data was filtered. For high-pass filtering, we applied a least squares FIR filter with filter order of 2,000, passband frequency of 0.5 Hz and stopband frequency of 0.45 Hz. For low-pass filtering, we employed a least squares filter with a filter order of 2,000, passband frequency of 25 Hz and stopband frequency of 27.5 Hz. Subsequently, EEG data was again downsampled to 128 Hz, and then normalized by subtracting the mean and dividing by the standard deviation. Next, the EEG data was epoched, centered around phoneme onsets, specifically from −0.2 s before phoneme onset to 0.6 s after onset. No baseline correction was applied during epoching. No further normalization was applied after epoching.

### Modelled features

The stimulus narrative was annotated for different properties at the phoneme level, namely 21 phonetic features, phoneme position relative to word onset and relative to word offset, as well as lexical entropy (Fig. 1*C*). To achieve this, an aligner (Duchateau et al., 2009) was used to create alignment files containing the identity and timing of each phoneme and each word of the stimulus. Then, we annotated phonetic features based on the multi-value feature system reported in King and Taylor (2000), which features voicing, manner of articulation, place of articulation, roundness and front-backness. Voicing indicates whether or not the vocal cords vibrate during speech production. Voicing has two features, voiced (e.g., /b/) and unvoiced (e.g., /*p*/). Manner describes how air flows through the articulators during speech production. We tested six manner features: nasal, fricative, occlusive and approximant for consonants, and short vowel and long vowel for vowels. Place refers to the positioning of the articulators (e.g., teeth, tongue, and lips) during speech production. We tested eight place features: dental, coronal, glottal, labial and velar for consonants, and low, mid, high for vowels. Roundness describes whether or not lips are rounded during pronunciation, thus consisting of two features, rounded (e.g., /w/) and unrounded (e.g., /f/). Front-backness refers to the position of the tongue in the mouth, entailing the features front, central, and back. We removed phonetic features that occurred in <5% of total phonemes in the stimulus, leading to the removal of three place features, i.e., dental, glottal, and low vowel. This led to 18 features that were used for further analysis.

#### Subset variables

To investigate whether phonemes are differentially encoded depending on their phoneme position within words and their lexical entropy, we subset phonemes based on these properties. Phoneme position relative to word onset was coded as 1 for the first phoneme within words, 2 for the second phoneme within words, etc. until the fifth position, and the opposite order for phoneme position relative to word offset. This means that there were less phoneme occurrences per position, as evidenced in Table 1. Concretely, this was executed by using the word onsets to chunk the phoneme onsets by word, then loop through the chunks and serialize the phoneme onsets within word.

**Table 1.** Phoneme occurrences for subset variables

Level	*n*	Meaning
*Phoneme position relative to word onset/offset*		
1	3,002	First/last phoneme within word
2	2,948	Second (to last) phoneme within word
3	2,199	Third (to last) phoneme within word
4	1,524	Fourth (to last) phoneme within word
5	1,187	Fifth (to last) phoneme within word
*Lexical entropy*		
1	3,427	Phonemes with low entropy
3	3,592	Phonemes with high entropy

Lexical entropy measures the level of competition among words that match the current phoneme input. For example, upon hearing the sounds /pl/, many potential words are activated (*n* = 999), indicating a high degree of competition. As additional phonemes are heard, the number of possible words diminishes (e.g., for /plu/, *n* = 162, and for /plur/, *n* = 19), resulting in a lower degree of competition. This competition level is calculated using the Shannon entropy of the words in the activated cohort (Gwilliams and Davis, 2022). The first phoneme of each word was excluded from this analysis. Entropy was calculated based on the SUBTLEX-NL database (Keuleers et al., 2010) and a custom pronunciation dictionary. Then we computed the 33rd and 66th percentiles to split entropy values into three equal parts and used the third with the lowest and the third with the highest values for the decoding analysis. Table 1 shows how many phoneme occurrences were present in the thirds.

### Decoding analysis

We decoded each phonetic feature from the 64-sensor EEG signal. All decoding was performed using one-versus-all logistic regression and fivefold cross-validation, using a temporal generalization (TG) approach. TG entails testing whether a temporal decoder trained on data at time *t* can accurately decode data at time *t* from a testing set (Fig. 1*E*). Instead of assessing decoding accuracy solely at the specific time point the model was trained on, we evaluated its accuracy across all possible train/test time combinations. TG is represented by a square decoding matrix of training time versus testing time (Fig. 1*E*). We assessed the decoding performance by computing the area under the curve (AUC) of the receiver-operating curve.

To assess for how long phonetic features can be decoded from brain data, we extracted the temporal decoders where train and test time were identical, i.e., the diagonal of the TG matrix, and extracted above chance time points. To assess for how long information captured at any given temporal decoder is maintained or generalized, we extracted the width of above chance clusters of the TG matrix. Additionally, to explore the dynamics of neural representations, we compared the mean duration of above chance generalization across temporal decoders to the duration of above chance temporal decoding (i.e., comparing the rows of the matrix to its diagonal). These metrics were examined within each participant and analyzed using second-level statistics across participants.

To further clarify how to interpret the diagonal of the TG matrix and the width of the pattern around the diagonal, we here elaborate:

- Decoding duration: the diagonal of the TG matrix, i.e., when the classifier is trained and tested on the same epoch time lag. This allows the classifier to learn the pattern of activity that optimally discriminates the feature of interest. Thus, every timepoint along the diagonal tells us whether the phonetic feature is decoded significantly above chance at that specific moment in time relative to phoneme onset. Here, we measure decoding duration by assessing for how long the diagonal decoding performance is significantly above chance-level.
- Maintenance/Generalization of the phonetic representation: the width of the TG matrix corresponds to the duration with which a classifier trained at a single time lag is able to read out information from preceding and subsequent time lags. If a phonetic representation is maintained within the same neural pattern for a long time, this indicates that the phonetic representation remains static within one configuration, or is passed slowly between neural configurations. Thus, the thicker the emerging pattern is around the diagonal (longer maintenance), the slower the speed of the evolving information between different neural configurations. And vice versa, the thinner the pattern is around the diagonal (shorter maintenance), the faster the speed of the evolving information between different neural configurations.

The number of subprocesses (or configurations) within neural processing of a phoneme is another factor that can also change, which allows the duration and generalization to alter independently.

To investigate which EEG sensors are most important for phonetic decoding, we decoded features from each sensor individually, thus passing the epoched timecourses of a given sensor to the decoder. This can tell us how much each sensor contributes across time to phonetic feature decoding.

Note on decoding/encoding terminology: we employ decoding analyses, using EEG data as input to predict speech features. Successfully decoding a feature implies its encoding in neural activity. We chose decoding because it (i) replicates the previous study’s approach (Gwilliams et al., 2022) and (ii) uses multivariate EEG data, offering robustness against noise and aggregating signals across channels (Brodbeck et al., 2021).

A second note concerns speech feature collinearity: because acoustic features like pitch variation correlate with phonetic features, our decoding results may reflect a mixture of both correlated acoustic properties and phonetic representations rather than purely phonetic representations.

#### Comparing performance between trial subsets

To assess decoding performance across theoretically interesting subsets of trials (such as start vs end of a word or high vs low entropy), we slightly changed our train/test cross-validation procedure. The classifier was trained using the full training dataset, while the test set was divided according to the different levels of interest (Table 1). We independently evaluated the model’s performance on each subset of the test data, yielding a timecourse or generalization matrix for each group of trials being analyzed.

### Statistical analysis

To compare TG matrices and the derived diagonals against chance-level, we used permutation-based statistics. This was performed on group-level average decoding performance using a one-sample permutation cluster test implemented using MNE Python (Gramfort et al., 2013). First, a *t*-value is calculated for every data point of the TG matrix. To determine statistical significance, the data undergoes 10,000 iterations of random permutation, wherein the signs of data points are randomly flipped. For each permutation, clusters of neighboring data points are identified, and the sum of *t*-values within these clusters is computed. This generates a null distribution of summed *t*-values from the permuted data. The significance of the observed effect is then assessed by comparing the observed cluster’s *t*-value sum to this null distribution. If the observed sum is greater than 95 % of sums from the null distribution, the effect is considered statistically significant.

To compare aphasia and control groups, we used the permutation cluster test as implemented in MNE Python (Gramfort et al., 2013). This test compares groups by first calculating differences at each data point of the TG matrices. It then shuffles data between groups 10,000 times to create a null distribution. Significant clusters, where differences are consistent, are identified by comparing observed values to those in the null distribution.

To test whether the TG matrix pattern is dynamically evolving (i.e., the diagonal is larger than the width of the decoding pattern), we (1) extracted the diagonal and horizontal (average across *y*-axis) timecourses per participant, (2) subtracted the diagonal from the horizontal timecourse for each participant, and (3) used a one-sample permutation cluster test to test whether this delta is significantly different from zero.

To analyze topographical differences of phonetic feature decoding between aphasia and control groups, we submitted the decoding performance of each sensor across subjects and groups to a mass-univariate independent samples *t*-test (Brodbeck, 2020). This implementation uses mass-univariate testing, where the null distribution is formed based on the maximum *t*-value across the entire sensor array of every permutation. The reported *p*-value is corrected for family-wise error rate.

## Results

### Replicating phonetic encoding dynamics in older adults with EEG data

**Figure 2.**
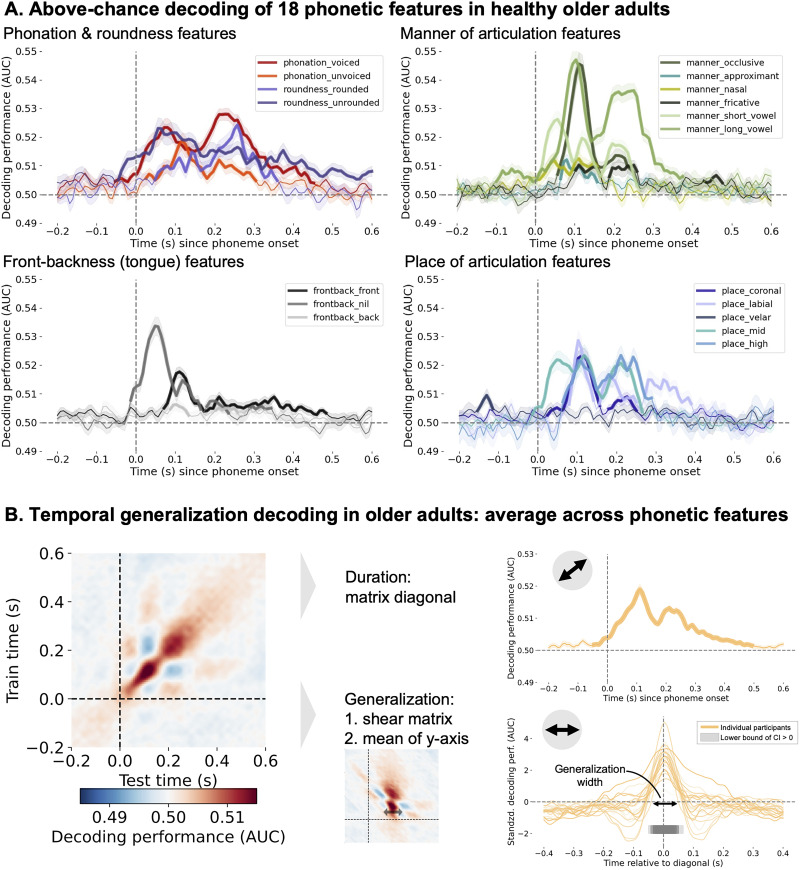
Phonetic feature decoding from EEG data in healthy older adults. ***A***, Above chance decoding of 18 phonetic features in healthy older adults. The features vary in decoding performance and over time. The thicker line segments indicate the time points where the features are significantly decoded above chance. ***B***, The temporal generalization (TG) approach in healthy older adults revealed that phonetic features are decodable above chance for 0.5 s (top right panel) and generalizable for 0.077 s (bottom right panel). The decoding duration corresponds to the diagonal of the TG matrix (left panel), thus the decoding performance when the decoder was trained on the same time slice as was used for testing the decoder. To quantify the generalization time, the TG matrix is reoriented and then averaged across the *y*-axis.

Gwilliams et al. (2022) found that the time window in which phonetic features were significantly decodable from MEG data was from 0.05 to 0.3 s after phoneme onset. Using the same approach, we computed a timecourse of decoding performance, reflecting to what extent the phonetic information is present in the EEG signal, for each of the 18 tested phonetic features. We found that all 18 phonetic features were decodable above chance using one-sample permutation cluster tests across subjects (cluster-forming threshold: *p* = 0.05; Table 2 and Fig. 2*A*). Due to variations in phoneme duration, such as between vowels and consonants, and the differing number of phoneme trials within certain phonetic feature categories, there is variability in decoding performance between features. Therefore, we focus on the decoding performance averaged across all features moving forward. When averaging across phonetic features, we observed above chance decoding from −0.04 to 0.49 s (*p* < 0.001) relative to phoneme onset for the control group (Fig. 2*B*, top right plot; Text S7). Thus, on average, phonetic features are decodable for a span of 0.53 s. Given that the median phoneme duration in the story was 0.070 s (min = 0.029 s; max = 0.359 s), this replicates the prior finding from younger adults (Gwilliams et al., 2022) that neural encoding of phonetic features long surpasses the duration of the phoneme itself. Results hereafter are all based on the average across phonetic features.

**Table 2.** Above chance decoding of phonetic features in healthy older adults

Phonetic feature	Significant time window (s)	Average decoding performance (AUC)	Average *t*-value	*p*-Value*
Voiced	−0.052 to 0.452	0.515	6.599	<0.001
Unvoiced	0.025 to 0.304	0.509	4.425	<0.001
Nasal	−0.013 to 0.165	0.508	3.659	<0.001
Fricative	0.102 to 0.165	0.508	3.465	0.003
Fricative	0.188 to 0.258	0.509	3.903	<0.001
Fricative	0.429 to 0.475	0.505	2.832	0.02
Occlusive	0.056 to 0.258	0.518	6.084	<0.001
Approximant	0.064 to 0.157	0.508	3.718	<0.001
Short vowel	−0.013 to 0.258	0.514	6.253	<0.001
Long vowel	−0.099 to 0.374	0.518	6.640	<0.001
Coronal	0.025 to 0.157	0.511	5.034	<0.001
Coronal	0.180 to 0.250	0.506	4.070	<0.001
Labial	0.079 to 0.141	0.516	4.889	<0.001
Labial	0.157 to 0.250	0.510	3.688	<0.001
Labial	0.273 to 0.390	0.511	3.963	<0.001
Velar	−0.153 to −0.114	0.506	3.517	0.007
Mid	−0.013 to 0.266	0.515	5.997	<0.001
High	0.064 to 0.289	0.516	4.759	<0.001
Rounded	0.048 to 0.359	0.511	4.232	<0.001
Unrounded	−0.044 to 0.6	0.512	5.026	<0.001
Front	0.071 to 0.483	0.506	4.208	<0.001
Central (nil)	−0.013 to 0.165	0.517	6.346	<0.001
Central (nil)	0.180 to 0.234	0.505	2.848	0.02
Central (nil)	0.289 to 0.343	0.505	3.076	0.01
Back	0.087 to 0.134	0.505	2.835	0.02
Back	0.203 to 0.258	0.504	3.170	0.007

* The cluster-forming threshold (critical *t*-value) was calculated as follows with a *p*-value of 0.05: thresh  =  scipy.stats.t.ppf ( 1−pval÷2, df); that is for a two-tailed *t*-test (df = degrees of freedom).

Sustained neural encoding implies simultaneous processing of multiple phonemes. Gwilliams et al. (2022) found that the brain encodes information from the preceding three phonemes, retaining their order by encoding time since onset. Using TG analysis, they revealed that phonetic information is maintained within a neural configuration for 0.08 s before evolving. This method provides insight into the speed of neural information encoding changes, demonstrating the dynamic nature of phonetic neural representation (Fig. 1*E*). Here, we investigate whether healthy older adults also show a dynamic processing scheme, given that this seems to be an important mechanism for orderly processing of phoneme sequences.

Using the TG decoding approach, we observed the train time by test time matrix shown in Figure 2*B* (left panel), wherein each data point represents the decoding performance. The width of the significant cluster in this matrix represents the generalization time of phonetic information in a given time point and spatial configuration (i.e., a decoder trained at time *t*). To extract the duration of generalization, we rolled the rows of the matrix such that the values along the diagonal become oriented at a given *x*-coordinate. We then average across the *y*-axis of the matrix, resulting in Figure 2*B* (right bottom panel). The width of the significant matrix cluster corresponds to 0.077 s in healthy older adults. We compared the phonetic encoding duration (0.53 s, the matrix diagonal, Fig. 2*B*, right top panel) to the generalization (0.077 s, the width of the reoriented matrix) using a one-sample permutation cluster test to confirm the dynamic evolution of phonetic encoding. We found a significant difference between −0.044 to 0.165 s [*t*(23) = −2.86, *p* < 0.001] and between 0.188 to 0.46 s [*t*(23) = −3.12, *p* < 0.001], confirming that phonetic information is encoded dynamically, evolving over time and space, in healthy older adults. The same analysis and figures for the aphasia group can be found in the supplementary information (Fig. S2 and Text S5).

### Phonetic encoding in individuals with aphasia

**Figure 3.**
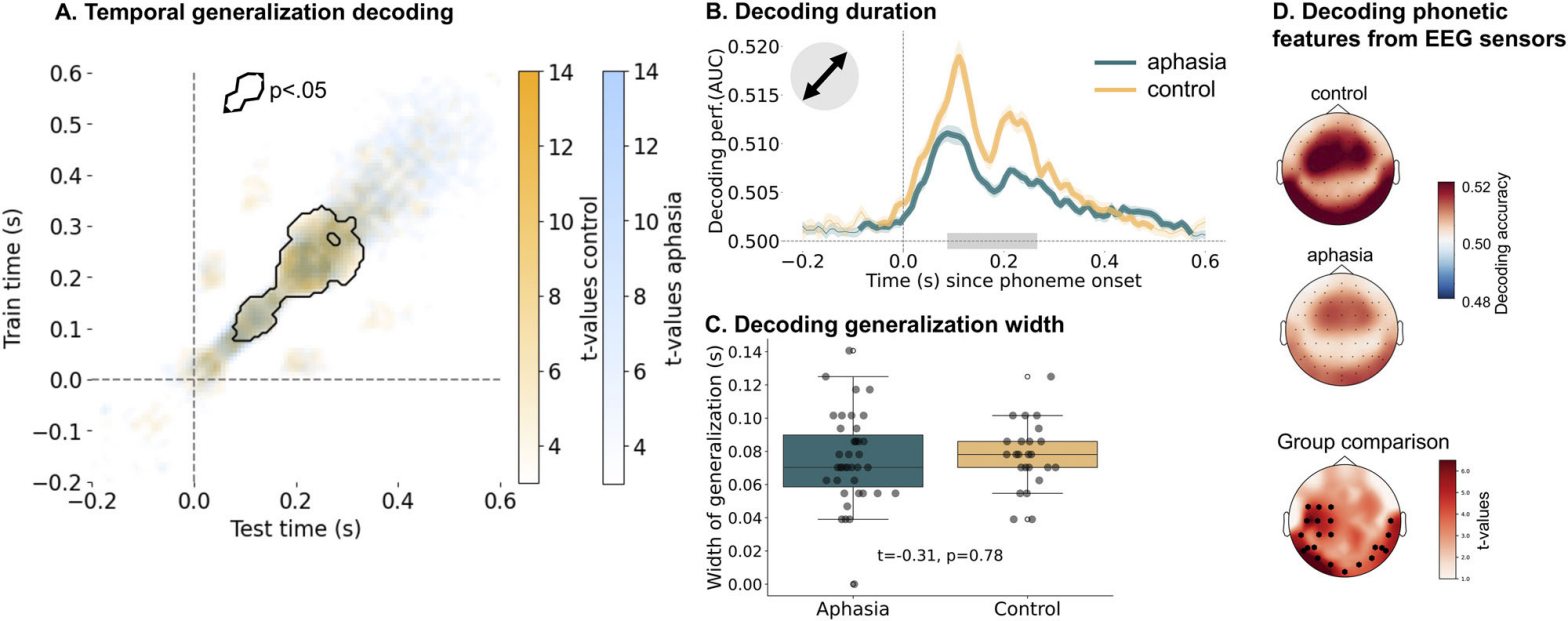
Comparing phonetic decoding between groups. ***A***, The TG matrices shown as t-values overlaid for the control (yellow) and aphasia (blue) group. To obtain this, a one-sample permutation cluster test was conducted for each group separately, which calculates a t-value for each data point in the TG matrix. The black contour indicates the cluster in which the groups significantly differed from each other. To achieve this, we conducted a permutation cluster test to compare groups. ***B***, A significant difference between groups was found from 0.08 to 0.25 s (as indicated by the gray bar) where the control group showed increased decoding performance. The bold parts of the lines indicate the time points where the features are significantly decoded above chance. ***C***, No significant difference in generalization was found between groups. For both groups, the encoded information was passed on to new neural ensembles every 0.07 s. ***D***, The top two topographies show how well phonetic features could be decoded for each of the 64 EEG sensors. The bottom topography shows the difference between both groups, specifically the t-values calculated using a mass-univariate independent samples t-test. The control group showed generally higher decoding, but especially in the 22 sensors marked in bold in the bottom topography.

IWA have been found to have impaired phoneme identification (Kries et al., 2023) and phonological processing (Blumstein, 1998). This led us to investigate the neural dynamics of phonetic encoding in aphasia in comparison to healthy controls. Regarding the TG analysis, we expected that the aphasia group would show a different encoding pattern than the healthy control group. We tested whether IWA would show shorter encoding duration, slower speed of evolution of encoding than controls, or a mix of both (Fig. 1*F*). Figure 3*A* shows the TG matrices for the aphasia and control groups overlaid. We conducted a permutation cluster test to compare matrices between groups and found a cluster in which the aphasia group showed significantly lower decoding performance than the control group (*p* < 0.001; contour in Fig. 3*A*).

The matrix diagonal shows phonetic feature decoding duration. We compared this duration between IWA and controls using a permutation cluster test. Results revealed significantly lower decoding accuracy in the aphasia group from 0.08 to 0.25 s post-phoneme onset (*p* < 0.001; Fig. 3*B*, S3 and Text S6). Based on the two observed peaks in the decoding timecourse, we additionally investigated whether the decoding performance at each peak correlates with different behavioral measures, within the aphasia group. We hypothesized that behavioral correlates of each peak could provide insight into the associated functional processes; however, no significant correlations were found between either peak and language- or cognition-related variables (Text S8).

To compare groups on how long phonetic information is maintained within a same temporal decoder and spatial configuration, we extracted the train time lags between 0 and 0.35 s, based on previous research (Gwilliams et al., 2022), and standardized the data (Fig. 3*A*). We then identified every participant’s width of generalization (“how *wide* is the peak?”) by (1) calculating the standard deviation across the individual time course, (2) computing a confidence interval for each value in the time course, and (3) extracting time points where the lower bound of the confidence interval is larger than 0, which equals the generalization width. Next, we conducted an independent samples *t*-test with 10k permutations to compare the generalization width between groups (Fig. 3*C*). No significant group difference was found (*t* = −0.31, *p* = 0.78), with average generalization time being 0.075 s in the aphasia group and 0.077 s in the control group. The speed of evolution of phonetic encoding across spatial configurations in IWA is thus highly overlapping with healthy controls.

Taken together, these results partially support the hypothesis in Figure 1*F* (top right panel): an altered duration of phonetic encoding in aphasia (i.e., weaker encoding after initial processing time), but no change to the speed with which phonetic information is passed between neural populations. However, the difference in duration of phonetic encoding that we observed is indirect, i.e., phonetic information is more weakly encoded in aphasia after initial processing time, a scenario that we did not fully capture in our hypotheses in Figure 1*F*.

Finally, to examine the spatial underpinning of these dynamic representations, we compared sensor-level topographical differences of phonetic feature decoding between aphasia and control groups (Fig. 3*D*). With a cluster-forming *p*-value threshold of *p* < 0.001, we identify a cluster of 22 EEG sensors (*p* < 0.001; Fig. 3*D*) over which IWA had lower decoding accuracy than healthy controls. The sensors primarily localized to left temporal and bilateral occipital sensor positions.

### Effects of lexical predictability on phonetic encoding

Given the observed difference in phonetic encoding duration between IWA and controls, we post hoc investigated potential causes. Previous research suggests lexical predictability influences phonetic encoding duration (Gwilliams et al., 2022). Lexical entropy, quantifying uncertainty about word identity, is typically higher for word-initial phonemes and lower for later phonemes (Gwilliams and Davis, 2022). We explored whether changes in phonetic encoding duration in aphasia relate to an altered interaction between phonetic encoding and lexical uncertainty. If so, encoding duration should correlate with lexical uncertainty: higher uncertainty would lead to longer phonetic information encoding.

**Figure 4.**
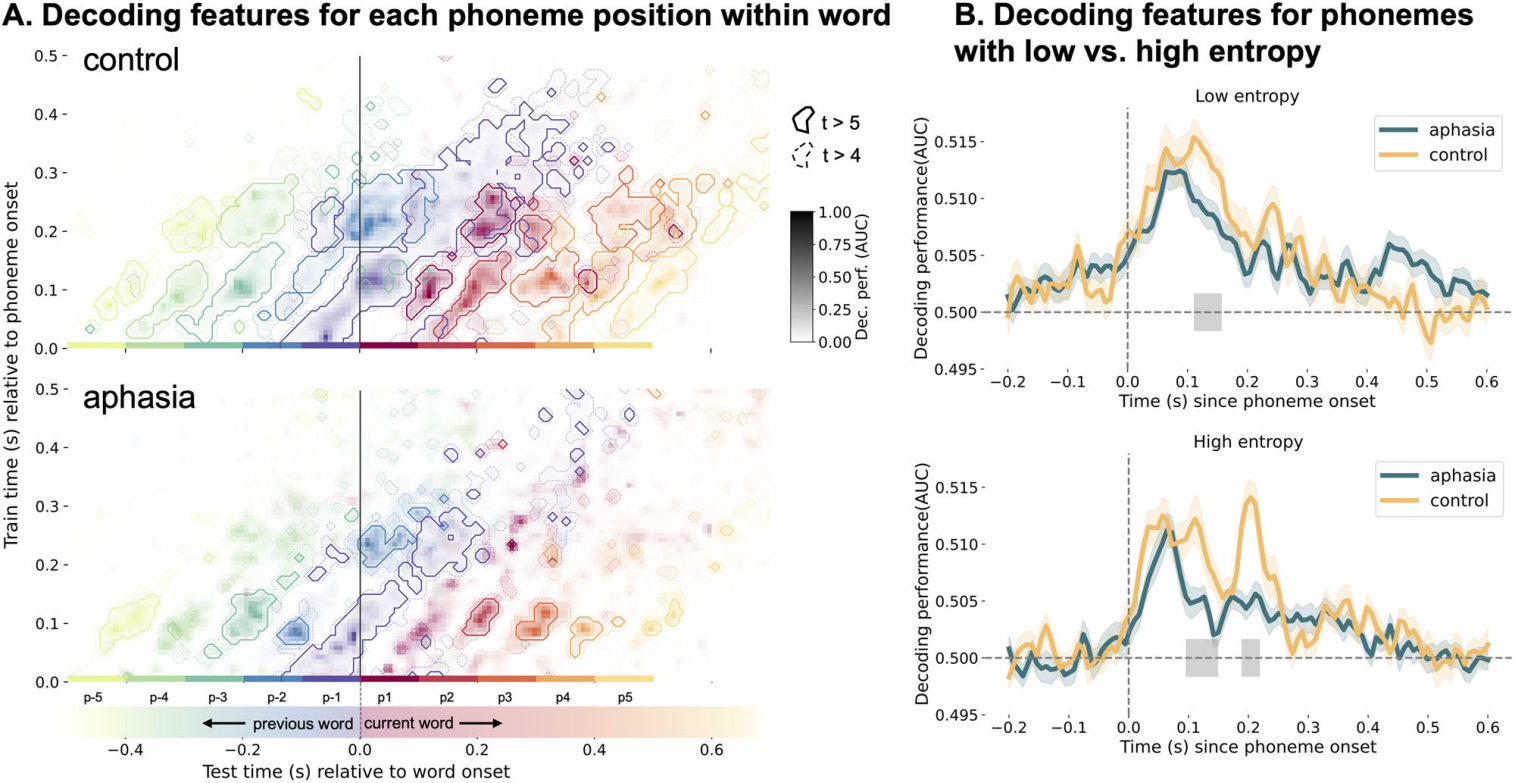
Testing the effect of phoneme position and lexical uncertainty on phonetic decoding in individuals with aphasia and controls. ***A***, The top plot shows temporal generalization (TG) decoding of phonetic features for each phoneme position within word separately. Each phoneme position is depicted in a different color. The bottom plot shows the same type of plot for the aphasia group. The TG matrices are shown as contour plots, where the contours frame *t*-values larger than 5 (solid lines) and 4 (dashed lines). The decoding strength is shown as gradient from light (lower decoding) to dark (higher decoding) in the respective color of each phoneme position. Phoneme positions where the decoding matrices significantly differed between groups are P1, P2, P3, P-2 and P-5. ***B***, We decoded phonetic features separately for phonemes with low and high lexical entropy. The top plot shows the difference between groups for low entropy phoneme decoding, the bottom plot shows the same graph for high entropy phoneme decoding. The gray bars indicate where the groups are significantly different from each other.

We conducted two analyses to investigate this; we subset (1) all phonemes according to phoneme position relative to word onset and offset and (2) phonemes according to lexical entropy. We compared groups on each phoneme position separately using a permutation cluster test, demonstrating a significant group difference for the first (*p* < 0.001), second (*p* = 0.02), and third (*p* = 0.03) phoneme positions, as well as the second to last (*p* < 0.01) and fifth to last (*p* = 0.016) phoneme positions (Fig. 4*A*). For the first phoneme relative to word onset, we observed a difference in reactivation, wherein only the control group seemed to maintain longer or “reactivate” the phonetic features in earlier temporal decoders, i.e., 0.1–0.25 s (Fig. S4 and Text S9). For the other phoneme positions, the group difference was located on or close to the TG matrix diagonal, thus displaying similar group comparison results as found when taking into account all phonemes, i.e., altered strength of encoding. We would like to point out that we might not have had enough phoneme trials beyond the second (and second to last) phoneme position (Table 1) to draw strong conclusions from this analysis.

Lexical entropy is one possible way to measure linguistic prediction (Gwilliams and Davis, 2022), which is represented within the language network (Shain et al., 2020), and given that the language network is damaged in aphasia, we expect the linguistic prediction mechanism to be disrupted. Therefore, we hypothesized that IWA would show no difference in phonetic decoding performance between phonemes with low and high entropy, but that healthy controls would show such a difference.

We compared groups in low and high entropy conditions separately using permutation cluster tests. In both conditions, controls showed higher decoding than the aphasia group between 0.1 and 0.15 s post-phoneme onset [low entropy: 0.110–0.157 s, average decoding: control = 0.013 and aphasia = 0.008, *t*(61) = 0.757, *p* = 0.042; high entropy: 0.095–0.149 s, average decoding: control = 0.009 and aphasia = 0.004, *t*(61) = 0.002, *p* = 0.011; Fig. 4*B*]. Additionally, for phonemes with high entropy only, the control group shows also higher decoding in a window from 0.188 to 0.219 s [average decoding: control = 0.012 and aphasia = 0.004, *t*(61) = 1.025, *p* = 0.002) after phoneme onset (Fig. 4*B*). Second, we tested the interaction between group and entropy condition by subtracting the low entropy condition from the high entropy condition (Fig. S5) for each group, subsequently conducting an independent samples *t*-test for each time lag and finally correcting *p*-values for multiple comparisons (false discovery rate). Based on findings from Gwilliams et al. (2022), we tested in a time window from 0.15 to 0.35 s. We found a significant interaction between group and entropy condition from 0.204 to 0.212 s after phoneme onset [*t*(61) = 2.145, *p* = 0.037], wherein the control group had higher decoding performance than the aphasia group in the high entropy condition only. This means that phonemes with higher lexical uncertainty are encoded for longer—an adaptive encoding duration mechanism found in previous work (Gwilliams et al., 2022). We replicated this finding in healthy older adults but found no evidence for the same processing occurring in people with aphasia.

**Figure 5.**
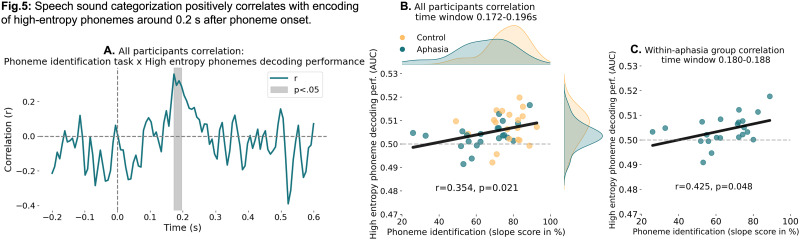
Speech sound categorization positively correlates with encoding of high entropy phonemes around 0.2 s after phoneme onset. ***A***, The high entropy phonetic feature decoding timecourse correlated with the phoneme identification task shows a significant positive correlation from 0.172 to 0.196 s after phoneme onset. Here the correlation coefficient timecourse is shown across groups. ***B***, Across groups correlation for significant time samples. Depicted are the individual data points as well as the distribution of values per group per measure. ***C***, Within-aphasia group correlation for significant time samples. Also see Text S10.

Given the significant difference in high entropy phoneme decoding between groups at 0.2 s post-speech-onset, we explored potential behavioral correlates within people with aphasia and across all participants. We tested correlations with behavioral acoustic, linguistic, cognitive and demographic variables in an exploratory way (no correction for multiple comparisons applied), which can be found in Text S10. Most importantly, we found a positive correlation between the phoneme identification task performance (data available in 22 IWA and 20 controls) and high entropy phoneme decoding performance around 0.19 s after phoneme onset (all participants: *r* = 0.354, *p* = 0.021; within-aphasia group: *r* = 0.425, *p* = 0.048; Fig. 5, Text S10). The phoneme identification task consisted of participants having to identify whether the sound they heard was a /bA/ or /dA/, evaluating how consistently people identify phonemes amid acoustic variability (Kries et al., 2023). This task addresses the “invariance problem”—the challenge of mapping variable speech sounds to discrete phonological categories. This significant correlation occurred around the same latency that the control group showed stronger phonetic feature encoding of high entropy phonemes than the aphasia group (Fig. 5).

## Discussion

We decoded phonetic features from EEG data during continuous story listening in 39 IWA and 24 age-matched (thus older) healthy controls. We found that (1) phonetic features can be robustly decoded from 25 min of EEG data in healthy older adults, (2) IWA have an altered phonetic encoding duration, strength, and topography compared to controls, and (3) the adaptive mechanism of phonetic encoding duration being contingent on lexical uncertainty seems to be disrupted in aphasia.

### Replicating phonetic encoding dynamics in older adults with EEG data

Robust encoding of phonetic features was reproduced in older adults using an EEG dataset (Fig. 2), which replicates a prior MEG study with younger adults (Gwilliams et al., 2022). This is an important result, given that the signal-to-noise ratio is higher for MEG than EEG data (Goldenholz et al., 2009; Ahlfors et al., 2010), and given the 3.7x difference in data size between studies, i.e., 2 h and 50,518 phonemes in Gwilliams et al. (2022) versus 25 min and 13,560 phonemes in the present study.

The fact that Gwilliams et al. (2022) used English stimuli and here we used Dutch stimuli may also introduce variation in results due to differing phonetic features and their statistical probabilities and frequency. Different phonetic features show slightly distinct decoding timecourses, e.g., short vowels have an earlier first peak than long vowels. All phonetic features were decodable above chance at some point throughout the timecourse. Pre-speech onset significant timepoints are likely due to co-articulation.

Finally, we also replicated the prior finding of dynamic coding of phonetic features in older adults. Average decoding across features was above chance for 0.53 s, whereas the duration with which the same neural pattern remained informative was only 0.07 s. Thus, in both younger and older adults, a dynamic neural pattern is used to encode phonetic features in continuous speech.

### Phonetic encoding in IWA

Comparing decoding duration between groups, we found that phonetic features were decoded less well in IWA than in controls (Fig. 3*B*). Early during processing (0–0.08 s), no significant difference between groups was found (Fig. 3*A*,*B*); the difference emerged from 0.08 to 0.25 s. IWA may initially process phonetic features but not keep the information encoded long enough to fully integrate it with higher-level processes (Sigman and Dehaene, 2005; Gilbert and Sigman, 2007; Gwilliams et al., 2018). Thus, the duration of encoding is indirectly affected in aphasia via lower encoding at later processing timepoints.

Comparing TG decoding performances of both groups (Figs. 3*A* and S3), it seems that the decoding performance is more diffuse, less concentrated in the aphasia than the control group. More diffuse cognitive representations have been found to be a part of the healthy aging process, a mechanism called dedifferentiation (Koen and Rugg, 2019). Dedifferentiation means a loss of functional specialization in neural structures. While both our control and aphasia samples were older adults (average 71 years), IWA might exhibit more pronounced dedifferentiation than controls. In fact, Purcell et al. (2019) found that increased local neural response differentiation was related to less severe language impairments. Similarly, Du et al. (2016) found that increased activation in frontal speech motor areas, despite more dedifferentiation, correlates with improved speech discrimination. Another possible explanation for more diffuse representations in aphasia is that EEG data is affected by the stroke lesion site being filled with cerebro-spinal fluid, which in turn impacts the signal-to-noise ratio over sensors closest to the lesion site (Park et al., 2016; Piastra et al., 2022).

Sensor-level decoding revealed similar topographical patterns across groups, but stronger encoding in controls, especially over left auditory sensors (Fig. 3*D*). This aligns with previous findings of decreased speech envelope encoding over similar sensors in this aphasia dataset (Kries et al., 2024). While structural changes in the left hemisphere may affect decoding accuracy (Fig. S1), functional changes in speech processing may also explain our finding. Mehraram et al. (2024) found weaker low-gamma-band left-hemispheric functional connectivity in IWA than in controls, and IWA that had increased delta-band connectivity within the left pre-frontal region also performed better on a picture naming task. De Clercq et al. (2024) demonstrated that lesions in the left middle temporal gyrus were most strongly linked to reduced encoding of speech acoustics. Our current analysis cannot distinguish between structural and functional changes. A control group of right-hemisphere stroke patients without aphasia could help clarify the nature of these effects.

Given that phonological impairments have been found in IWA such as mixing up sequence order or phoneme misidentification (Blumstein, 1998), we expected to see a more square-shaped TG matrix (Fig. 1*E*), meaning that IWA would pass on information at a slower pace between neural populations, creating more representational overlap between phonemes of a sequence. However, the neural pattern evolves at the same rate for both groups (Fig. 3). Specifically, the neural pattern completes a full evolutionary cycle every 0.07 s. This implies that phonological impairments in IWA are not related to not being able to keep apart phonetic representations of neighboring phonemes.

### Effects of lexical predictability on phonetic encoding

Our third research question emerged post hoc, to understand what might underlie changes in phonetic decoding duration/strength in aphasia. At a few phoneme positions, TG decoding matrices differed between groups (Fig. 4). Most markedly, word-initial phonemes’ features—generally the phoneme with the highest uncertainty about lexical identity—were maintained longer/”reactivated” in decoders relatively early after phoneme onset in healthy controls, but not in IWA. This suggests that IWA may not keep phonetic features of word-initial phonemes encoded for long enough, such that integration with higher levels of processing may be hampered. Age differences between this study (older adults) and Gwilliams et al. (2022; younger adults) may explain discrepancies in word-initial phoneme pre-activation results. Older adults might process predictive cues differently (Gillis et al., 2023), possibly reactivating and maintaining phonetic features (Fig. S4 and Text S9) rather than pre-activating them. However, other methodological differences between studies limit direct comparisons. Future research could examine these hypotheses in younger and older adults.

We also assessed how lexical entropy affects phonetic encoding in IWA and controls. Controls, but not IWA, encoded phonemes with high lexical uncertainty for a longer period of time than phonemes with low lexical uncertainty. Our findings in healthy controls (older adults) align with Gwilliams et al. (2022)’s results in young adults, suggesting that the ability to flexibly adjust encoding duration based on lexical uncertainty may be crucial. IWA lack this adaptive encoding mechanism, which might thus lead to language processing impairments. IWA appear unable to encode phoneme properties for a sufficient duration to resolve lexical identity. This shows that integration between different processing levels might be maladaptive in aphasia.

The variability in phonetic feature decoding of high entropy phonemes around 0.2 s after phoneme onset was associated with behaviorally assessed phoneme identification (Fig. 5, Text S10). Individuals who performed better at the phoneme identification task also showed stronger phonetic encoding when word identity was uncertain. This finding further supports that maintaining phonetic features long enough to resolve word identity is an important mechanism to support successful comprehension. Moreover, this mechanism has potential as a neurobiological marker of aphasia.

### Applying the hierarchical dynamic coding framework to aphasia

Gwilliams et al. (2022) demonstrated that the duration for which phonetic features are maintained in the same neural population (0.08 s) match with and is contingent on the duration of the phoneme input (0.08 s). Here, the duration of maintaining phonetic information (0.077 s) is similar to the median phoneme duration in the stimulus (0.070 s), thus is in line with previous findings. Recently, Gwilliams et al. (2025) extended TG analysis to higher linguistic levels, revealing HDC as a general sensory-cognitive processing principle. Given our findings of weaker, more diffuse phonetic feature encoding in IWA, this pattern might extend to other language processing levels in aphasia. If neurotypical healthy adults with intact speech comprehension show a specific coding pattern, while individuals with a language impairment show a different pattern, then maybe the neurotypical coding pattern is conditional for successful speech comprehension. It is possible that a certain spatio-temporal concentration in neural encoding of speech features (e.g., phonetic features) is necessary to efficiently integrate information with other language representation levels.

### Caveats

Limitations include (1) a heterogeneous and small sample of 39 IWA. Future studies should aim to reproduce findings with larger samples or more narrowly-recruited samples, longer recordings, and extend analysis to word- or phrase-level representations. (2) Group analyses may mask individual behavioral-neural relationships. (3) The lesion site and its effect on neural recordings pose a problem for more detailed interpretation of results. (4) Our analysis approach is not reliable at the individual level, at least not with the relatively limited amount of data available. (5) We cannot attribute our results to purely phonetic feature decoding, due to speech feature collinearity. Other acoustic factors such as pitch and amplitude variation could also play a role in the impaired link between lower-level sensory and word-level processing in aphasia.

### Conclusion

Phonetic features are robustly encoded in EEG responses of older adults. The primary marker of aphasia was altered strength of phonetic encoding after initial processing, rather than slower evolution of the neural representation. Moreover, phonemes with high uncertainty about word identity were encoded longer in controls than in IWA, indicating that encoding phonetic information until word identity is resolved may be a crucial mechanism for successful speech comprehension. These results suggest that speech impairments in aphasia may be driven by difficulties maintaining lower-level information long enough to recognize lexical items.

## Data Availability

The datasets generated during and/or analyzed during the current study and code are available publicly on Open Science Framework: https://osf.io/whc6y/overview?view_only=d6a8c1b569b54ed889de8ad8e8596866.
